# Mental Health, Substance Use, and Tuberculosis Preventive Therapy in People With HIV: A Prospective Cohort Study

**DOI:** 10.1093/ofid/ofaf303

**Published:** 2025-06-04

**Authors:** Ann E Johnson, Lucy Chimoyi, Sheela Shenoi, Marie A Brault, Laura Forastiere, Salome Charalambous, Violet Chihota, J Lucian Davis

**Affiliations:** Yale School of Medicine, New Haven, Connecticut, USA; Epidemiology of Microbial Diseases, Yale School of Public Health, New Haven, Connecticut, USA; Implementation Research Division, The Aurum Institute, Parktown, Johannesburg, South Africa; School of Public Health, University of the Witwatersrand, Parktown, Johannesburg, South Africa; Epidemiology of Microbial Diseases, Yale School of Public Health, New Haven, Connecticut, USA; Section of Infectious Diseases, Department of Internal Medicine, Yale School of Medicine, New Haven, Connecticut, USA; Department of Population Health, New York University Grossman School of Medicine, New York, New York, USA; Department of Biostatistics, Yale School of Public Health, New Haven, Connecticut, USA; Implementation Research Division, The Aurum Institute, Parktown, Johannesburg, South Africa; School of Public Health, University of the Witwatersrand, Parktown, Johannesburg, South Africa; Implementation Research Division, The Aurum Institute, Parktown, Johannesburg, South Africa; School of Public Health, University of the Witwatersrand, Parktown, Johannesburg, South Africa; Division of Infectious Diseases, Department of Medicine, Vanderbilt University School of Medicine, Nashville, Tennessee, USA; Epidemiology of Microbial Diseases, Yale School of Public Health, New Haven, Connecticut, USA; Section of Pulmonary, Critical Care, and Sleep Medicine, Department of Internal Medicine, Yale School of Medicine, New Haven, Connecticut, USA

**Keywords:** depression, HIV, medication adherence, South Africa, tuberculosis

## Abstract

**Background:**

Because of the association of mental health and substance use disorders with higher HIV mortality and decreased retention in care, we investigated their frequency and impact on tuberculosis preventive therapy (TPT) adherence and completion among people with HIV (PWHIV) initiating TPT.

**Methods:**

We conducted a prospective, longitudinal cohort study with a nested mixed methods study in 2 Johannesburg, South Africa, facilities. Participants were PWHIV on antiretroviral therapy initiating TPT between August and December 2023. We measured TPT adherence and completion with electronic medication boxes. We used validated tools to measure symptoms of anxiety, depression, alcohol use, and other substance use at enrollment and 12 weeks. We constructed multivariable regression models to determine associations of these variables with TPT adherence and completion, adjusting for age, sex, and time on antiretroviral therapy. We interviewed participants about mental health and experiences with TPT and analyzed responses using deductive content analysis.

**Results:**

Among 224 PWHIV, 111 (50%) completed TPT. Eighty-one (36%) screened positive for depression symptoms and 63 (28%) for anxiety symptoms. Seventy-six (34%) reported unhealthy alcohol use and 60 (27%) tobacco use. Using multivariable models adjusting for confounders, only depression symptoms were significantly and negatively associated with adherence (9% fewer doses; 95% confidence interval, .4–16; *P* = .032) and completion (odds ratio, 0.48; 95% confidence interval, .26–.90; *P* = .021). Participant narratives highlighted the negative influence of mental health on adherence and the need for social and psychological support services.

**Conclusions:**

Symptoms of depression, anxiety, unhealthy alcohol use, and tobacco use were common among PWHIV initiating TPT. Depression symptoms were strongly and independently associated with TPT nonadherence and noncompletion.

## Teaser

Depression, anxiety, unhealthy alcohol use, and tobacco use were common among people living with HIV initiating tuberculosis preventive therapy in South Africa. Depression symptoms were strongly and independently associated with tuberculosis preventive therapy nonadherence and noncompletion.

Tuberculosis (TB) remains the leading cause of death for people with HIV (PWHIV) [1] despite improvements in TB screening, diagnosis, and prevention [2]. Antiretroviral therapy (ART) can reduce TB incidence by 66% among PWHIV [3], and combining TB preventive therapy (TPT) and ART reduces TB incidence by 76% [4, 5] and long-term mortality by 37% [6]. Because of TPT's effectiveness, strengthening TPT delivery to PWHIV is critical to achieving the World Health Organization End TB Strategy of reducing TB incidence and mortality [6, 7]. However, TPT uptake among PWHIV is low, with only 34% of eligible PWHIV newly enrolled in HIV care initiating TPT in 2022 [8]. Reports of TPT completion among PWHIV vary between 25% and 95% [9]. This variability partly reflects differences in adherence measurement but also individual and structural factors that impede TPT adherence and completion, including cognitive impairment [10], food insecurity [11], the type and duration of the regimen prescribed [9], unhealthy alcohol use [12], and mental health disorders [13]. Mental health and substance use disorders are common among PWHIV and are associated with higher HIV mortality and lower ART adherence and retention [13].

South Africa has the world's largest HIV epidemic of >7.7 million PWHIV and is 1 of the world's highest TB burden countries [8, 14]. To combat the TB-HIV syndemic, South Africa introduced and began scaling up a novel 3-month TPT regimen, 12 doses of weekly isoniazid–rifapentine (3HP) in 2021 [15]. 3HP is a safer, better-tolerated option than the traditional daily 6- or 12-month regimens [16]. In addition to being at high risk of TB, about 30% of South Africans with HIV have an alcohol use disorder [17], 38% have moderate or severe depression, and 53% experience regular psychological distress or anxiety [18]. Although evidence about the impact of substance use and mental health conditions on 3HP outcomes is scarce, these conditions are associated with worse ART outcomes among PWHIV [19, 20] and lower TPT adherence for people with TB infection [21, 22]. The objectives of this study are to determine the frequency of mental health and substance use disorders among PWHIV initiating 3HP and the impact of these comorbidities on 3HP adherence and completion.

## METHODS

### Study Design and Setting

We conducted a prospective, longitudinal cohort study with a nested mixed-methods study enrolling PWHIV initiating 3HP to determine the frequency of comorbid mental health and substance use disorders and their influence on TPT adherence and completion. From August to December 2023, we enrolled participants at 2 Community Health Centers (CHCs) in townships outside Johannesburg, South Africa. Each CHC diagnoses around 80 patients with HIV monthly. These government-funded CHCs provide free diagnosis and treatment for HIV and TB and began offering 3HP in 2021. South Africa's 2023 guidelines recommend 3HP for all adult PWHIV within 2 weeks of HIV diagnosis, except for those not virally suppressed or beginning a dolutegravir-containing ART regimen [23].

### Procedures and Measures

Trained research nurses screened consecutive PWHIV initiating 3HP for study eligibility using the following inclusion criteria: (1) age ≥18 years, (2) initiating or already taking ART, (3) initiating 3HP, and (4) willing to receive 3HP in a medication-event reminder monitor (MERM) box. After obtaining written informed consent, we collected demographic and clinical information from study participants, including HIV diagnosis date, time on ART, and employment.

We used validated instruments to assess depression symptoms (Patient Health Questionnaire-9 [PHQ-9]) [24], anxiety symptoms (Generalized Anxiety Disorder [GAD-7]) [25], and alcohol use (Alcohol Use Disorders Identification Test–Consumption [AUDIT-C]) [26 , 27]. The PHQ-9 score ranges from 0 to 27, with higher scores indicating more severe depression symptoms. Severity is graded as mild (5–9), moderate (10–14), moderately severe (15–19), or severe (20–27). The GAD-7 score ranges from 0 to 21, with higher scores indicating more severe anxiety symptoms. Severity is graded as mild (5–9), moderate (10–14) or severe (15–21). The AUDIT-C score ranges from 0 to 12, with higher scores indicating a greater risk of alcohol-related problems. Unhealthy alcohol use is defined as a score ≥4 for men or ≥3 for women. We assessed tobacco, opioid, cannabis, and other substance use with the Alcohol, Smoking, and Substance Involvement Screening Test (ASSIST) [28]. We referred participants screening positive for moderate or severe depression (PHQ-9 ≥10) or anxiety (GAD-7 ≥ 10) symptoms, or suicidality to the clinic social worker and psychologist for management and support.

At this initial visit, nurses dispensed 3HP in an EvriMED1000 MERM (Wisepill Technologies, Cape Town, South Africa), with reminders disabled. We used the MERM to assess the primary outcome TPT completion (≥11 3HP doses within 12 weeks) and the secondary outcome TPT adherence (proportion of 12 total doses taken). We considered a single MERM box opening within each calendar day as a proxy for a dose taken. We monitored battery levels and recalled participants for recharge for levels ≤20%.

Participants returned the MERM during a routine clinical visit 12 weeks after 3HP initiation. We called each participant 1 week before their 3-month enrollment date for the sole purpose of scheduling a follow-up visit. We screened for unhealthy alcohol use and anxiety and depression symptoms at both the enrollment and the 3-month follow-up visits. We screened for other substance use only at the enrollment visit. We collected self-reported adherence and completion using an adapted Simplified Medication Adherence Questionnaire [29] aligned 3HP's weekly schedule. As an additional adherence measure, we performed the IsoScreen test (GFC Diagnostics, Oxfordshire, UK), a urine assay for isoniazid metabolites [30], on a subset of participants whose follow-up visit occurred after institutional review board approval of this additional procedure. We collected all data in REDCap, a secure electronic data-capture software [31].

### Statistical Analysis

We estimated that a sample size of 224 participants would provide 80% power at a significance level of 0.05 to detect a ≥35% change in TPT completion, which is our primary outcome, assuming TPT completion rates of 30% with symptoms of depression, anxiety, or substance use and 65% without these comorbidities and accounting for an expected 20% lost to follow-up.

Participants were classified as having depression symptoms if their PHQ-9 scores were ≥5 and as having anxiety symptoms if their GAD-7 scores were ≥5. These thresholds, which correspond to mild symptoms in each category, are associated with adverse health outcomes [32, 33]. We summarized demographic and clinical characteristics using proportions for categorical variables and medians and interquartile ranges for continuous variables. We imputed missing data using median values. We assessed MERM accuracy against urine IsoScreen by calculating the positive predictive value of box opening for biomaker-defined adherence. We constructed a multivariable logistic regression model of the association of symptoms of depression, anxiety, unhealthy alcohol use, and tobacco use with 3HP completion, adjusting for previously reported potential confounders, including age [12], sex [34], years since initiating ART [34], employment [35], and marriage [21]. To assess adherence predictors, we built a multivariable β-regression model with the same variables, adjusting for the same confounders. We assessed for collinearity using Pearson's correlation, goodness-of-fit with the Hosmer-Lemeshow test, and model stability using ordinary nonparametric bootstrapping.

Using logistic regression models, we assessed interactions and determined the association of each combination with 3HP completion. We conducted a variety of sensitivity analyses, including examining the effects of each exposure individually (ie, depression symptoms, anxiety symptoms, alcohol use, tobacco use) and of having symptoms of any comorbidity (including depression, anxiety, or unhealthy alcohol, tobacco, cannabis, cocaine, or sedative use), adjusting for the same potential confounders (age, sex, years since initiating ART, employment, and marriage). We analyzed all data using R (RStudio, Boston, MA) [36].

### Mixed-methods Analysis

We conducted an explanatory sequential mixed methods study at the 12-week follow-up visit to understand participant perspectives on the influence of substance use and mental health on 3HP adherence and completion. We interviewed a consecutive subset of participants using a 9-question interview guide (Supplementary Appendix A) informed by the baseline mental health and substance use results.

Trained research assistants conducted the interviews in English, Zulu, and Sepedi, based on the participant's preference. The 2 female and 2 male researchers had training in qualitative interviewing techniques and over 5 years of experience conducting TB-HIV research. Research assistants transcribed and translated participant responses into electronic REDCap data entry forms in real time. A female American doctoral student (A. E. J.) conducted deductive content analysis in MAXQDA 12 (VERBI, Berlin, Germany) [37], using a codebook that included depression symptoms [20], anxiety symptoms [18], unhealthy alcohol use [19], time on ART [34], and marriage [21], all selected from the literature as key factors in treatment adherence and completion. Codes were refined into themes through iterative discussion. After A. E. J. coded the interview responses, the full team of 3 analysts refined the themes based on a review of the coded data and through iterative discussions until they achieved consensus. We integrated our quantitative and qualitative results using a joint display to summarize the impact of these factors on 3HP adherence and completion. The joint display includes quotations illustrating and contextualizing quantitative relationships for participants who completed or did not complete 3HP.

### Human Subjects

The University of the Witwatersrand Human Research Ethics Committee (#210212) and the Yale University Human Investigation Committee (#2000035520) approved the study protocol.

## RESULTS

### Characteristics of the Study Population

From August to December 2023, we screened 228 consecutive PWHIV initiating 3HP and enrolled 224 participants (Supplementary Appendix Figure A1). We excluded 3 individuals who declined 3HP and 1 who declined the MERM box. All returned for their 12-week follow-up visit. The final 62 (28%) consecutive participants provided urine for isoniazid metabolite testing and completed a qualitative interview.

Median age was 44 years (interquartile range [IQR] 37–51), and 136 (61%) were female. Median time on ART was 7.2 years (IQR 3.3–10.4) (Table 1). A total of 204 (91%) completed secondary school, and 120 (54%) were employed full-time.

**Table 1.** Sociodemographic and Clinical Characteristics of Study Participants

Characteristic Median (IQR), or n (%)	Overall Study (n = 224)	Qualitative Substudy (n = 62)
Age (y)	44 (37–51)	46 (39–54)
Female sex	136 (61%)	37 (60%)
Married	94 (42%)	27 (44%)
Secondary education or higher	121 (54%)	32 (52%)
Employed^a^	120 (54%)	31 (50%)
Years since HIV diagnosis	8 (3.4–11.1)	9 (4.2–11.9)
Years on ART	7 (3.3–10.4)	9 (4.2–10.9)
Any comorbidity^b^	155 (69%)	45 (73%)
Depression symptoms^c^	81 (36%)	23 (37%)
Anxiety symptoms^d^	63 (28%)	22 (35%)
Unhealthy alcohol use^e^	76 (34%)	20 (32%)
Tobacco use^f^	60 (27%)	21 (34%)
Cannabis use^f^	18 (8%)	12 (19%)

Abbreviations: ART, antiretroviral therapy; ASSIST, Alcohol, Smoking and Substance Involvement Screening Test; AUDIT-C, Alcohol Use Disorders Identification Test-Concise; GAD-7, Generalized Anxiety Disorder-7 Questionnaire; IQR, interquartile range; PHQ-9, Patient Health Questionnaire-9.

^a^Excluding informally employed and those receiving government assistance.

^b^At least 1 of depression or anxiety symptoms, or unhealthy alcohol, tobacco, cannabis, cocaine, or sedative use.

^c^PHQ-9 ≥ 5.

^d^GAD-7 ≥ 5.

^e^AUDIT-C ≥ 4(men), ≥3(women).

^f^ASSIST ≥4.

Of the participants, 155 (69%) had at least 1 mental health or substance use comorbidity, including symptoms of depression, anxiety, or unhealthy substance use. Eighty-one (36%) participants screened positive for depression symptoms, including 75 (33%) with mild depression symptoms (PHQ-9 score 5–9), 5 (2%) with moderate depression symptoms (PHQ-9 10–14), and 1 (<1%) with moderately severe depression symptoms (PHQ-9 score 15–19). Sixty-three (28%) screened positive for anxiety symptoms, including 61 (21%) with mild anxiety symptoms (GAD-7 score 5–9) and 2 (1%) with moderate anxiety symptoms (GAD-7 score 10–14). Seventy-six (34%) reported unhealthy alcohol use (AUDIT-C score ≥4 for men, ≥3 for women) and 60 (27%) reported tobacco use. Eighteen (8%) reported cannabis use, 3 (1%) sedative use, and 1 (0.4%) cocaine use, all in the moderate-risk category (ASSIST score 4–26). Zero participants reported using amphetamines, inhalants, hallucinogens, or opioids. At the 3-month follow-up visits, participants’ numeric scores on the PHQ-9, GAD-7, and AUDIT-C scales were similar to their scores at the enrollment visit. Thirty-eight participants had 3 comorbidities and 91 had 2. Pearson's correlation between depression symptoms and alcohol use was 0.07, between anxiety symptoms and alcohol −0.01, and between symptoms of depression and anxiety 0.23 (Supplementary Appendix Figure A2).

### Completion Outcomes

As measured by MERM, 111 (50%) participants completed 3HP. As measured by self-report, 190 (85%) participants completed 3HP, but only 97 (51%) of these were confirmed to have completed 3HP by MERM.

### Bivariate and Multivariable Analysis

In bivariate analysis, the odds of 3HP completion were substantially and significantly lower among those with any comorbidity (odds ratio [OR], 0.52; 95% confidence interval [CI], .29–.92; *P* = .025), those with depression symptoms (OR, 0.45; 95% CI, .26–.79; *P* = .005) and those with tobacco use (OR, 0.54; 95% CI, .29–.98; *P* = .044). Completion of 3HP was also substantially but not statistically significantly lower among those with anxiety symptoms (OR, 0.63; 95% CI, .35–1.13; *P* = .12) and those with unhealthy alcohol use (OR, 0.69; 95% CI, .39–1.20; *P* = .19; Table 2).

**Table 2.** Unadjusted Associations Between 3HP Completion and Predictors

Characteristic n (%)	3HP Completion (n = 111)	3HP Noncompletion (n = 113)	OR (95% CI)	*P* Value
Age (y)				
18–35	14 (42%)	19 (58%)	—	Reference
35–50	62 (51%)	60 (49%)	1.40 (.65–3.05)	.39
51+	35 (51%)	34 (49%)	1.40 (.61–3.22)	.43
Female sex	70 (51%)	66 (49%)	1.22 (.71–2.08)	.48
Married	47 (50%)	47 (50%)	1.03 (.61–1.75)	.91
Employed^a^	60 (50%)	60 (50%)	1.04 (.61–1.76)	.89
Years on ART				
<3	25 (46%)	29 (54%)	—	Reference
3–10	49 (45%)	60 (55%)	0.95 (.49–1.82)	.87
10+	37 (61%)	24 (39%)	1.79 (.85–3.75)	.12
Any comorbidity^b^	69 (45%)	86 (55%)	0.52 (.29–.92)	.025
Depression symptoms^c^	30 (37%)	51 (63%)	0.45 (.26–.79)	.005
Anxiety symptoms^d^	26 (41%)	37 (59%)	0.63 (.35–1.13)	.12
Unhealthy alcohol use^e^	33 (43%)	43 (57%)	0.69 (.39–1.20)	.19
Tobacco use^f^	23 (38%)	37 (62%)	0.54 (.29–.98)	.044
Cannabis use^f^	8 (44%)	10 (56%)	0.80 (.30–2.11)	.65

Abbreviations: 3HP, 12-dose weekly tuberculosis preventive therapy regimen of isoniazid–rifapentine; ART, antiretroviral therapy; ASSIST, Alcohol, Smoking and Substance Involvement Screening Test; AUDIT-C, Alcohol Use Disorders Identification Test-Concise; GAD-7, Generalized Anxiety Disorder-7 Questionnaire; PHQ-9, Patient Health Questionnaire-9.

^a^Excluding informally employed and those receiving government assistance.

^b^At least 1 of depression or anxiety symptoms, or unhealthy alcohol, tobacco, cannabis, cocaine, or sedative use.

^c^PHQ-9 ≥ 5.

^d^GAD-7 ≥ 5.

^e^AUDIT-C ≥ 4(men), ≥3(women).

^f^ASSIST ≥4.

In the primary multivariable model that was adjusted for alcohol use, tobacco use, anxiety symptoms, age, sex, marital status, years since initiating ART, and employment status, only depression symptoms (OR, 0.48; 95% CI, .26–.90; *P* = .021) were significantly associated with 3HP completion (Table 3). The model was a good fit to the data (*P* = .86 by Hosmer-Lemeshow). Bootstrapping analysis confirmed the model's stability.

**Table 3.** Adjusted Associations Between 3HP Completion and Mental Health and Substance Use Predictors

Variable	Adjusted OR (95% CI)*	Adjusted *P* Value*
Depression symptoms^a^	0.48 (.26–.90)	.021
Anxiety symptoms^b^	0.69 (.36–1.30)	.25
Unhealthy alcohol use^c^	0.84 (.44–1.60)	.60
Tobacco use^d^	0.66 (.32–1.36)	.26

Abbreviations: 3HP, 12-dose weekly tuberculosis preventive therapy regimen of isoniazid–rifapentine; ASSIST, Alcohol, Smoking and Substance Involvement Screening Test; AUDIT-C, Alcohol Use Disorders Identification Test-Concise; CI, confidence interval; GAD-7, Generalized Anxiety Disorder-7 Questionnaire; OR, odds ratio; PHQ-9, Patient Health Questionnaire-9.

^*^Adjusted for sex, age, years since initiating antiretroviral therapy, main source of household income, and marriage.

^a^PHQ-9 ≥ 5.

^b^GAD-7 ≥ 5.

^c^AUDIT-C ≥ 4(men), ≥3(women).

^d^ASSIST ≥4.

### Sensitivity Analysis

Multivariable models examining the effects of individual exposures (depression symptoms, anxiety symptoms, unhealthy alcohol use, and tobacco use) on 3HP noncompletion, adjusted for sex, age, marital status, years since initiating ART, and employment, did not differ significantly from the multivariable model containing all variables (Supplementary Appendix Table A1).

### Adherence Analysis

Participants completed a median of 10 doses (IQR 6–12, 83% of required doses; Supplementary Appendix Figure A3). In a multivariable β-regression model adjusting for age, sex, marriage, employment, anxiety, unhealthy alcohol use, and tobacco use, depression symptoms were associated with 9% fewer 3HP doses by MERM (95% CI, .4–16; *P* = .032). Thirty-four of the total 62 (55%) participants providing urine for isoniazid metabolite testing were reported by MERM to have taken 3HP within the past 72 hours, and 30 (positive predictive value = 88%) of these tested positive for isoniazid metabolites in urine, indicating high agreement between MERM-measured adherence and a biometric reference standard.

### Mixed Methods Study Findings

We interviewed 62 participants for the mixed-methods substudy. Their characteristics were similar to the overall sample. Thematic analysis identified themes including mental health and treatment persistence, alcohol use as a coping mechanism, the influence of ART adherence habits on 3HP adherence, and the role of family in 3HP adherence and completion. Each theme is described further below.

### Mental Health and Treatment Persistence

Participants described feeling sad or hopeless, which impacted their ability to take 3HP. Although they did not explicitly mention “depression” or “anxiety,” these feelings fit the diagnostic criteria for depression and may be associated with a lack of motivation to engage in self-care [10]. One participant with depression symptoms described how feelings of fatigue and futility interrupted adherence, leading to noncompletion (Table 4). Another participant described how mental health challenges affected her, but she ultimately found the cognitive and emotional resources to overcome her doubts and complete 3HP.

**Table 4.** Joint Display of Risk Factors and Participant Quotes About 3HP Completion and Noncompletion

Factor	OR (95% CI)*	*P* Value*	Theme	3HP Completers	3HP Noncompleters
Depression symptoms^a^	0.45 (.26–.79)	.005	Mental health and treatment persistence	“When I felt down/sad I would question if the medication was necessary but like I said, I forced myself for the benefit of my health.” —54-year-old female	“I was feeling really tired and really just wanted to give up. I skipped 3HP that week because I felt that there was no need for it. I honestly do not do anything [to manage my mental health]. The social worker has tried to help me with some issues.” —34-year-old female with depression
Anxiety symptoms^b^	0.63 (.35–1.13)	.12	Mental health and treatment persistence	“When I overthink about my life and I am stressed I struggle to sleep sometimes and as a result I am tired in the morning and do not want to do anything but sit. It happens when I am overthinking about life. I normally talk to my mother, she makes me feel better.” —39-year-old female with depression	“It has been the same. I still worry and cry a lot but I am coping. Sometimes I just feel like I do not want to socialize with anyone so I lock myself up in the house to avoid people.” —34-year-old female with depression, anxiety, and tobacco use
Unhealthy alcohol use^c^	0.69 (.39–1.20)	.19	Alcohol use as a coping mechanism	“After taking 3HP I feel like not having beer.” —46-year-old male with anxiety, unhealthy alcohol use, and tobacco use	“I felt like I could give up,” and that as a result she felt “in a way that I should consume a lot of alcohol.” —37-year-old female with depression, anxiety, and unhealthy alcohol use “I prefer drinking a few beers to calm down and get into a happy mood.” —36-year-old male with depression, unhealthy alcohol use, and tobacco use
Time on ART (y)	1.06 (1.00–1.12)	.067	Influence of ART adherence habits on 3HP adherence	“I didn't have any special routine. I just took the 3HP at the same time as my ART. Only difference was the 3HP was on Thursdays only.” —41-year-old female with anxiety and unhealthy alcohol use	“I tried to take it at the same time on Tuesdays but I think there were times I took earlier or a bit later.” —34-year-old female with depression, anxiety, and tobacco use
Married	1.03 (.61–1.75)	.91	Role of family in 3HP adherence and completion	“My child reminded me all the time to take my medication.” —39-year-old female with depression	“At home, they would discourage me from taking so many pills but I kept taking them.” —47-year-old female with anxiety

Abbreviations: 3HP, 12-dose weekly tuberculosis preventive therapy regimen of isoniazid–rifapentine; ART, antiretroviral therapy; AUDIT-C, Alcohol Use Disorders Identification Test-Concise; GAD-7, Generalized Anxiety Disorder-7 Questionnaire; PHQ-9, Patient Health Questionnaire-9.

^*^From bivariate model of predictor and 3HP completion.

^a^PHQ-9 ≥ 5.

^b^GAD-7 ≥ 5.

^c^AUDIT-C ≥ 4(men), ≥3(women).

Participants reported anxiety symptoms such as stress, worry, and “overthinking,” which sometimes affected adherence. One participant described feeling worried and crying, which led her to avoid others and not complete treatment. Her experience shows how anxiety can interfere with 3HP completion.

### Alcohol Use as a Coping Mechanism

Some participants reported using alcohol to cope with mental health challenges. One participant described wanting to stop 3HP treatment and drink heavily (Table 4). Another participant described alcohol use to cope with depression symptoms. Despite this qualitative evidence, our quantitative analysis found no significant relationship between unhealthy alcohol use and 3HP noncompletion or adherence. We did not find collinearity or a significant interaction between alcohol and depression symptoms.

### The Influence of ART Adherence Habits on 3HP Adherence

ART adherence habits emerged as a facilitator of 3HP completion. One participant who had been on ART for more than a decade found it easy to integrate 3HP into her ART adherence routine. This corroborated the strong quantitative association between time on ART and 3HP completion. In addition, participants with difficulty establishing an ART routine also struggled to take their 3HP consistently.

### The Role of Family in 3HP Adherence and Completion

Family emerged as another important source of support or hindrance. A participant who completed treatment described how her child's support helped mitigate anxiety's impact on adherence. Spousal support was repeatedly mentioned as important for 3HP completion.

However, some family members made treatment completion more challenging by discouraging preventive therapy. For example, family members expressed concern that “so many pills” would damage the participant's health.

## DISCUSSION

In this prospective cohort study of PWHIV initiating 3HP, symptoms of depression, anxiety, unhealthy alcohol use, and tobacco use were extremely common, each affecting about one third of the study population and associated with lower 3HP completion rates, which were already low at 50%. After adjusting for clinical and demographic factors, we found that only depression symptoms were significantly associated with reduced adherence and completion, with individuals with depression symptoms completing 3HP only 37% of the time. Qualitative findings supported these results, as participants described the negative impact of depression symptoms on 3HP adherence, the maladaptive use of alcohol as a coping mechanism, and the potentially mitigating influence of ART adherence routines and family support. These findings suggest a need to integrate the management of depression, adverse effects, and adherence support into 3HP delivery to improve completion rates. Improving 3HP completion is crucial due to realizing the substantial mortality benefit of TB prevention among PWHIV.

Mental health and substance use frequency in this study were comparable to the findings of other studies of PWHIV in South Africa [20, 28]. Previous research shows that depression is associated with nonadherence. For example, Shearer and colleagues reported depression symptoms in 7% of virologically suppressed PWHIV and 40% of those with recent viral load elevations [20], comparable to our findings that 46% 3HP noncompleters had depression symptoms, versus 27% of 3HP completers. Similarly, a 2020 study in South Africa found that adolescents with HIV who screened positive for depression symptoms were more likely to have an unsuppressed viral load than adolescents without depression symptoms [24]. A meta-analysis found that depression during TB treatment was associated with higher mortality and more frequent loss of follow-up [38]. These papers suggest a variety of mechanisms through which depression symptoms may impact adherence, including by affecting neurocognition and associated decision-making and executive functioning abilities [10, 38]. Our qualitative evidence supports this, as participants linked melancholic symptoms to poor adherence.

Although many studies have found a significant association between alcohol use and reduced adherence, including to daily isoniazid preventive therapy for PWHIV [12], to TB disease treatment [39], and to different daliy TPT regimens among people with TB infection [21, 22], we were unable to confirm such an association in our study. Although the effect size in our study was large, we could not exclude an association due to chance, perhaps because of insufficient sample size.

Several factors may explain the lack of association between substance use or anxiety symptoms and TPT outcomes in our study. One possibility is that these factors are genuinely not associated in our population because of differences from other populations where such associations have been observed. Another explanation could be measurement error, as anticipated stigma surrounding substance use and mental health may have led to underreporting. Finally, an association may exist, but our study may have been underpowered to detect a small effect.

Our findings and prior studies suggest the need to integrate interventions addressing mental health and substance use into HIV care and TB prevention. Despite the high prevalence of mental health and substance use disorders, access to treatment of these conditions in sub-Saharan Africa is limited [40]. Our qualitative findings revealed that family support helped some participants persist with weekly treatment despite mental health challenges. Future research should adapt evidence-based strategies like family adherence–partner programs, which involve training a family member to provide regular adherence support throughout treatment, to foster family support for TB treatment adherence [41]. Future research should also further investigate the qualitative findings of alcohol use as a coping mechanism for depression and the influence of ART adherence habits on 3HP adherence. Other promising emerging interventions for mental health and substance use include incorporating screening, referrals, and therapeutic interventions into HIV care [42, 43].

During qualitative data collection, participants provided more detailed accounts of their emotional states and the manifestations of their depression symptoms than during quantitative data collection. This aligns with existing research on HIV and depression that suggests that depression screening and management must be sensitive to the language people use to describe their feelings and adaptable to the cultural context in which they are applied [44, 45].

A major strength of this study is our use of validated tools for identifying mental health symptoms and substance use conditions , as well as 2 objective adherence measures. A second study strength is that the longitudinal study design allowed us to measure anxiety and depression symptoms and alcohol use both at baseline and follow-up. The fact that we found similar mental health symptoms and substance use scores at both time points,suggests that our baseline measures likely reflect participants’ exposures throughout their time taking TPT. Finally, we used qualitative methods to contextualize findings and integrated them with quantitative results through mixed methods analysis.

Our study had several limitations. We may have preferentially referred experienced clients for TPT, as indicated by the long median ART duration. Substance use and mental health impacts may differ for recent ART initiators. MERM boxes, although objective, may overestimate adherence if participants do not take medications after opening their MERM boxes or underestimate adherence if medications are obtained elsewhere. Although we disabled the reminder features, other aspects of MERMs, including the convenient storage they offer, may have favorably influenced adherence. We used the mild-or-greater severity scoring thresholds for depression and anxiety symptoms, whereas prior studies typically uses moderate-or-greater thresholds. Nevertheless, we found that even mild anxiety and depression symptoms had strong unadjusted associations with nonadherence and that depression symptoms had a strong adjusted association with nonadherence to and noncompletion of TB prevetive treatment. This reinforces previous studies that have found associations between mild anxiety or mild depression symptoms and health outcomes [32, 33, 46–48]. Direct transcription of interviews limited their length and depth. Finally, we conducted the study in 2 health centers in a single South African city, limiting the generalizability of our findings to other settings.

In conclusion, symptoms of depression and anxiety, and alcohol and tobacco use were common among PWHIV initiating 3HP in South Africa. Depression symptoms were strongly and independently associated with 3HP nonadherence and noncompletion. Improving integrated mental health services and HIV care in South Africa requires rigorous epidemiological data on the prevalence, severity, and impact of these conditions. Future studies should explore targeted interventions for comorbid depression among PWHIV initiating 3HP to help increase adherence and completion.

## Supplementary Material

ofaf303_Supplementary_Data

## Supplementary Data

Supplementary materials are available at *Open Forum Infectious Diseases* online. Consisting of data provided by the authors to benefit the reader, the posted materials are not copyedited and are the sole responsibility of the authors, so questions or comments should be addressed to the corresponding author.
