# Supplementary material for: Mental Health, Substance Use, and Tuberculosis Preventive Therapy in People With HIV: A Prospective Cohort Study
Source: Open Forum Infect Dis. 2025 Jun 4;12(6):ofaf303. doi: 10.1093/ofid/ofaf303 (PMC12188208; doi:10.1093/ofid/ofaf303)
Supplement: ofaf303_Supplementary_Data [file ofaf303_supplementary_data.zip › A1_TABLE_Revised.docx]

TABLE A1. Adjusted associations between 3HP completion and predictors

|  | Depression Model  OR (95% CI) | *p* value | Anxiety Model  OR (95% CI) | *p* value | Alcohol Model  OR (95% CI) | *p* value | Tobacco Model  OR (95% CI) | *p* value | Any Comorbidity Model  OR (95% CI) | *p* value |
| --- | --- | --- | --- | --- | --- | --- | --- | --- | --- | --- |
| Age (Years) | 0.99 (0.96-1.02) | 0.37 | 0.99 (0.96-1.02) | 0.55 | 0.99 (0.96-1.02) | 0.37 | 0.99 (0.96-1.02) | 0.47 | 0.99 (0.96-1.02) | 0.36 |
| Female Sex | 1.22 (0.69-2.15) | 0.49 | 1.25 (0.72-2.19) | 0.43 | 1.10 (0.61-1.95) | 0.75 | 0.94 (0.51-1.72) | 0.83 | 1.05 (0.59-1.86) | 0.87 |
| Married | 1.06 (0.61-1.87) | 0.83 | 1.00 (0.57-1.74) | 0.99 | 1.06 (0.61-1.85) | 0.83 | 1.02 (0.58-1.77) | 0.95 | 1.10 (0.63-1.92) | 0.74 |
| Employed^a^ | 0.82 (0.46-1.44) | 0.49 | 1.01 (0.59-1.74) | 0.96 | 1.00 (0.58-1.72) | 0.99 | 0.97 (0.56-1.68) | 0.93 | 0.90 (0.51-1.57) | 0.71 |
| Years on ART | 1.07 (1.00-1.14) | 0.049 | 1.07 (1.01-1.14) | 0.035 | 1.06 (1.00-1.13) | 0.066 | 1.06 (1.00-1.14) | 0.053 | 1.06 (1.00-1.13) | 0.069 |
| Depression symptoms^b^ | 0.42 (0.23-0.75) | 0.004 |  |  |  |  |  |  |  |  |
| Anxiety symptoms^c^ |  |  | 0.56 (0.30-1.03) | 0.066 |  |  |  |  |  |  |
| Unhealthy Alcohol Use^d^ |  |  |  |  | 0.73 (0.39-1.33) | 0.30 |  |  |  |  |
| Tobacco Use^e^ |  |  |  |  |  |  | 0.52 (0.26-1.02) | 0.059 |  |  |
| Any Comorbidity^f^ |  |  |  |  |  |  |  |  | 0.52 (0.28-0.95) | 0.037 |
| **Legend:** ^a^Excluding informally employed and those receiving government assistance; ^b^PHQ-9 ≥5; ^c^GAD-7 ≥5; ^d^AUDIT-C ≥4(men), ≥3(women); ^e^ASSIST ≥4; ^f^At least one of depression or anxiety symptoms, or unhealthy alcohol, tobacco, cannabis, cocaine, or sedative use | | | | | | | | | | |
| **Abbreviations:** 3HP: 12-dose weekly tuberculosis preventive therapy regimen of isoniazid–rifapentine; ASSIST: Alcohol, Smoking and Substance Involvement Screening Test; AUDIT-C: Alcohol Use Disorders Identification Test-Concise; GAD-7: Generalized Anxiety Disorder-7 Questionnaire; PHQ-9: Patient Health Questionnaire-9 | | | | | | | | | | |
