# Supplementary material for: Mental Health, Substance Use, and Tuberculosis Preventive Therapy in People With HIV: A Prospective Cohort Study
Source: Open Forum Infect Dis. 2025 Jun 4;12(6):ofaf303. doi: 10.1093/ofid/ofaf303 (PMC12188208; doi:10.1093/ofid/ofaf303)
Supplement: ofaf303_Supplementary_Data [file ofaf303_supplementary_data.zip › APPENDIX_A_Participant Interview Guide.docx]

Appendix A. Participant Interview Guide

1. Please describe your weekly routine when it comes to taking your 3HP.
2. How do you feel about taking 3HP?
3. Please describe any occasions when your mental health impacted your routine with taking 3HP.
4. Tell me about your mental health over the past 3 months.
   1. How does your mental health affect your behaviors?
   2. How do you manage your mental health?
5. Please tell me about support or resources you used to help you stay consistent with your 3HP treatment.
6. What additional forms of support or resources do you wish you had to assist you in adhering to your 3HP treatment?
7. Is there anything else you think I should know?
