# Supplementary material for: Mental Health, Substance Use, and Tuberculosis Preventive Therapy in People With HIV: A Prospective Cohort Study
Source: Open Forum Infect Dis. 2025 Jun 4;12(6):ofaf303. doi: 10.1093/ofid/ofaf303 (PMC12188208; doi:10.1093/ofid/ofaf303)
Supplement: ofaf303_Supplementary_Data [file ofaf303_supplementary_data.zip › MH_Appendix_B.docx]

Consistency of Comorbidity Scores

We evaluated the consistency of comorbidity scores between the initial and follow-up visits with a paired sample analysis. We quantified the magnitude of change in scores with Cohen’s d, which provides an effect size by dividing the mean difference in paired scores by the standard deviations of those differences.

The comparison of comorbidity scores between the initial and follow-up visits revealed negligible changes. The effect size for the change in anxiety scores was small (Cohen’s d: 0.109), as was the case for depression (Cohen’s d: 0.035), and problematic alcohol use (Cohen’s d: 0.052). Six participants did not have a repeat score for the follow-up visit, and those were excluded from the Cohen’s d calculation.

Consistent vs. Inconsistent Responses between Self-Report and MERM

Comparing participants with inconsistent responses between self-report and MERM to those with consistent responses using Fisher’s exact test, we observed higher proportions with depression symptoms (41% vs. 32%, p=0.16), anxiety symptoms (34% vs. 23%, p=0.10), unhealthy alcohol use (39% vs. 29%, p=0.12), and tobacco use (32% vs. 22%, p=0.13) in the inconsistent group. However, the differences were small and may be attributable to chance given the small sample sizes.
