# Supplementary material for: Mental Health, Substance Use, and Tuberculosis Preventive Therapy in People With HIV: A Prospective Cohort Study
Source: Open Forum Infect Dis. 2025 Jun 4;12(6):ofaf303. doi: 10.1093/ofid/ofaf303 (PMC12188208; doi:10.1093/ofid/ofaf303)
Supplement: ofaf303_Supplementary_Data [file ofaf303_supplementary_data.zip › Revised_A1_FIGURE_caption.docx]

Figure A1. CONSORT Diagram

This CONSORT diagram shows the flow of participants through the screening, eligibility, follow-up, and analysis phases of the study.

Alt text: Flow diagram depicting 228 people screened, 4 people not eligible, 224 people eligible, and 224 participant’s data analyzed.
