# Supplementary material for: Mental Health, Substance Use, and Tuberculosis Preventive Therapy in People With HIV: A Prospective Cohort Study
Source: Open Forum Infect Dis. 2025 Jun 4;12(6):ofaf303. doi: 10.1093/ofid/ofaf303 (PMC12188208; doi:10.1093/ofid/ofaf303)
Supplement: ofaf303_Supplementary_Data [file ofaf303_supplementary_data.zip › revised_A2_FIGURE_caption.docx]

Figure A2. Correlation matrix of mental health and substance use conditions in study participants

The Pearson correlation coefficients (PCC) between anxiety and depression symptoms, alcohol use, and tobacco use are shown. The PCC range from -1 to 1, with positive values indicating a positive relationship and negative values indicating a negative relationship between variables. The color intensity corresponds to the strength of the correlation, with stronger positive correlations indicated by darker shades of green and stronger negative correlations by darker shades of red.

Alt text: This is a correlation matrix where darker green colors indicate a correlation closer to 1 and darker red colors indicate a correlation closer to -1. None of the correlations are red. The correlations between alcohol use and tobacco, and between anxiety and depression symptoms are the darkest green.
