# Supplementary material for: Mental Health, Substance Use, and Tuberculosis Preventive Therapy in People With HIV: A Prospective Cohort Study
Source: Open Forum Infect Dis. 2025 Jun 4;12(6):ofaf303. doi: 10.1093/ofid/ofaf303 (PMC12188208; doi:10.1093/ofid/ofaf303)
Supplement: ofaf303_Supplementary_Data [file ofaf303_supplementary_data.zip › revised_A3_FIGURE_caption.docx]

Figure A3. Distribution of 3HP Doses Completed by Participants

This figure shows the number of 3HP doses taken out of the 12 total doses on the x-axis, and the proportion of the total cohort who took each total number of doses on the y-axis. Dose amounts to the right of the red dashed line are considered a completed course of 3HP.

Alt text: Bar graph comparing the number of 3HP doses taken on the X axis to the proportion of the total cohort on the Y axis. A red dashed line to the left of 11 doses indicates that participants with 11 doses or more completed their 3HP course. There is a wide spread of the number of doses people took, with doses concentrated at 11 and 12.
