# Supplementary figures and images for: Mental Health, Substance Use, and Tuberculosis Preventive Therapy in People With HIV: A Prospective Cohort Study
Source: Open Forum Infect Dis. 2025 Jun 4;12(6):ofaf303. doi: 10.1093/ofid/ofaf303 (PMC12188208; doi:10.1093/ofid/ofaf303)

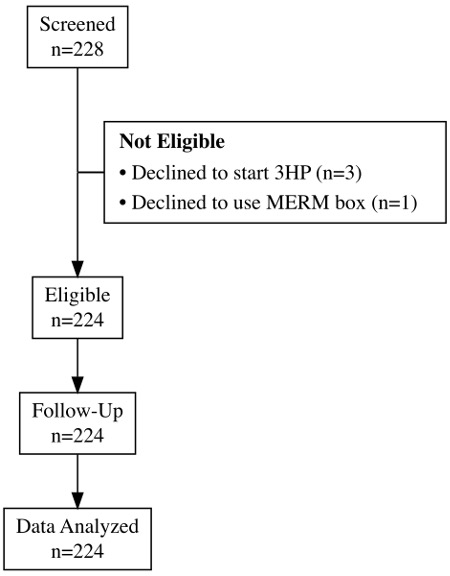

Supplement: ofaf303_Supplementary_Data [file ofaf303_supplementary_data.zip › A1_FIGURE.tiff]

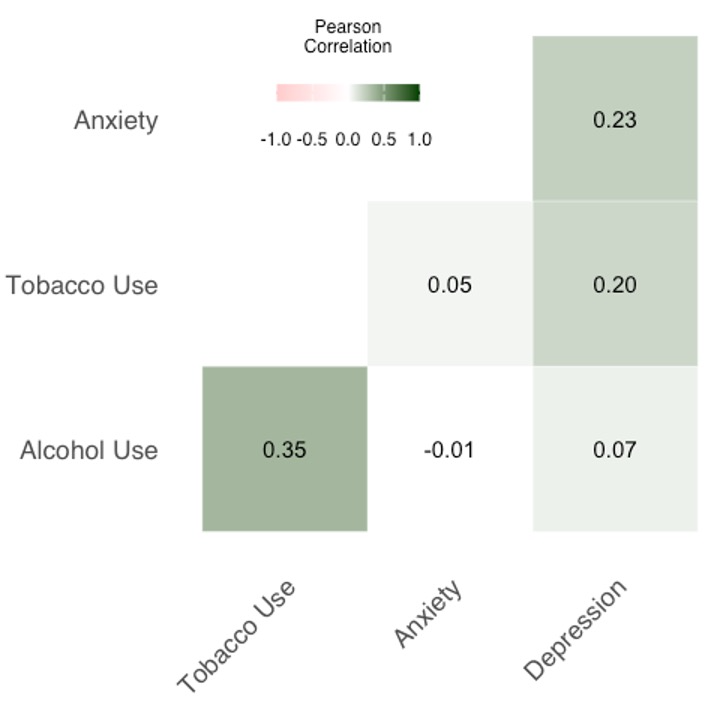

Supplement: ofaf303_Supplementary_Data [file ofaf303_supplementary_data.zip › A2_FIGURE.tiff]

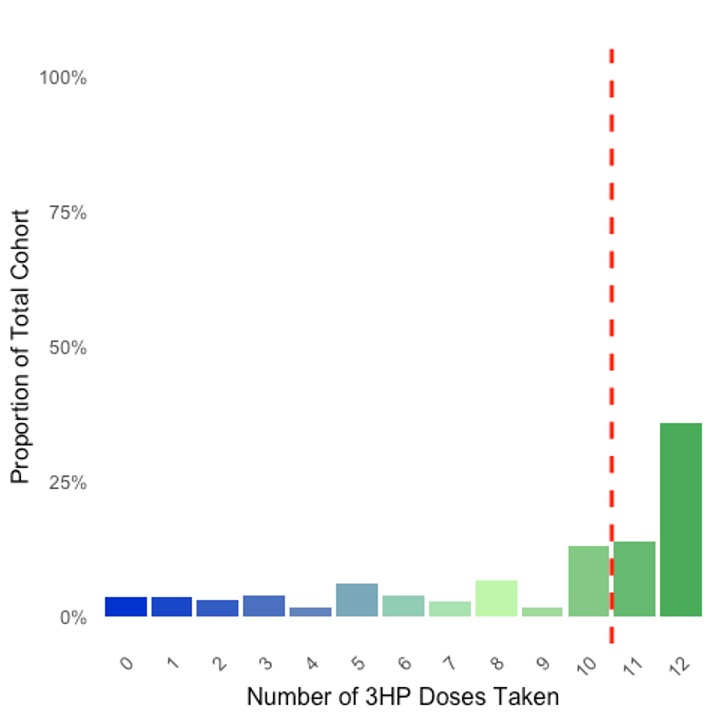

Supplement: ofaf303_Supplementary_Data [file ofaf303_supplementary_data.zip › A3_FIGURE.tiff]
